# The Impact of Heterogeneous Thresholds on Social Contagion with Multiple Initiators

**DOI:** 10.1371/journal.pone.0143020

**Published:** 2015-11-16

**Authors:** Panagiotis D. Karampourniotis, Sameet Sreenivasan, Boleslaw K. Szymanski, Gyorgy Korniss

**Affiliations:** 1 Department of Physics, Applied Physics, and Astronomy, Rensselaer Polytechnic Institute, 110 8^th^ Street, Troy, NY, 12180-3590, United States of America; 2 Social Cognitive Networks Academic Research Center, Rensselaer Polytechnic Institute, 110 8^th^ Street, Troy, NY, 12180-3590, United States of America; 3 Department of Computer Science, Rensselaer Polytechnic Institute, 110 8^th^ Street, Troy, NY, 12180-3590, United States of America; 4 Społeczna Akademia Nauk, Łódź, Poland; IFIMAR, UNMdP-CONICET, ARGENTINA

## Abstract

The threshold model is a simple but classic model of contagion spreading in complex social systems. To capture the complex nature of social influencing we investigate numerically and analytically the transition in the behavior of threshold-limited cascades in the presence of multiple initiators as the distribution of thresholds is varied between the two extreme cases of identical thresholds and a uniform distribution. We accomplish this by employing a truncated normal distribution of the nodes’ thresholds and observe a non-monotonic change in the cascade size as we vary the standard deviation. Further, for a sufficiently large spread in the threshold distribution, the tipping-point behavior of the social influencing process disappears and is replaced by a smooth crossover governed by the size of initiator set. We demonstrate that for a given size of the initiator set, there is a specific variance of the threshold distribution for which an opinion spreads optimally. Furthermore, in the case of synthetic graphs we show that the spread asymptotically becomes independent of the system size, and that global cascades can arise just by the addition of a single node to the initiator set.

## Introduction

The technological breakthroughs of the 21st century have strongly contributed to the emergence of network science, a multidisciplinary science with applications in many scientific fields and technologies. Several sociological opinion diffusion models first introduced in the middle of 20th century are now being thoroughly studied, while variations of these classical models have been introduced. Most of these models are based on social reinforcement, where simple rules based on the interaction of individuals with their respective nearest neighbors govern individual opinion evolution. The macroscopic outcome of these rules is a cascade of nodes switching opinions [1–9]. We focus our study on one of the classic models of social influencing, the Threshold Model (TM). The TM is a binary opinion spread model first introduced by Granovetter [2] to model collective behavior socially driven by peer pressure. Under the TM a node adopts a new opinion only when the fraction of its nearest neighbors possessing that opinion is larger than an assigned threshold, which represents the resistance of the node to peer pressure [3]. Although the microscopic rule of opinion adoption in the TM is simple, the collective behavior that arises is complex and non-linear. The resulting spread size depends on a large set of parameters, such as the network structure (e.g., clustering) [7, 10–13], the size of the initially active nodes (initiators), the selection strategy of the initiators, and the distribution of threshold values among nodes of the network. The first thorough investigation of the TM was made by Watts [5], who examined the effect of one randomly selected initiator on the cascade size. Gleeson and Cahalane [14–16], on the other hand, determined analytically the cascade size for varying initiator sizes (or fractions) for the infinite system size. Recent investigations of the TM by Karimi and Holme [17] and Michalski et al. [18] also considered the impact of temporal networks on contagion cascades. Very recently, Ruan et al. [19] studied the effects of “immune” individuals (those who resist adopting the new idea indefinitely) and external influencing (e.g., by mass media or advertisements) in the TM.

An important problem in generalized models for social and biological contagion [20–22] is to optimize the set of initiators, i.e., for a fixed cost (seed size), find the set of initiators giving rise to the largest cascade, or alternatively, find the minimum size seed set required to activate the entire network [23]. As far as selection strategies are concerned, Kempe et al. [24] showed that the optimization problem of selecting the most influential nodes in any directed weighted graph with uniform random selection of thresholds is NP-hard. They also suggested a greedy algorithm [24], where each new initiator is selected based on the maximum spread it can cause, which unfortunately resulted in low efficiency of the algorithm. Chen et al. [25] designed a scalable algorithm (LDAG) which is based on the properties of directed acyclic graphs. Recently, Lim et al. [26] introduced a new node-level measure of influence, called cascade centrality (based on the size of the cascade resulting from the node being the only initiator), which may guide the selection of multiple initiators. Closely related to these studies and of practical interest is to find a set of initiators (not necessarily the smallest) in a scalable fashion that guarantees that the entire network will ultimately turn active, triggered by these initiators [27]. Their method was inspired by the *k*-shell decomposition of the network [28], which itself can be an effective heuristic for selecting initiators in a broad class of models for the spreading of social or biological contagion [21].

Singh et al. [10] studied the effect in the TM of varying the fraction of initiators on the cascade size for various basic heuristic selection strategies when each node has identical threshold in the network. They showed that there is a critical fraction of initiators (“tipping point”) at which a sharp (discontinuous) phase transition occurs from small to large cascades in Erdős-Rényi (ER) graphs [29]. This phase transition is apparent for the random, *k*-shell, and degree-ranked selection strategies, which are listed in the increasing order of their performance. These findings, in particular, the emergence of the discontinuous transition, were analogous to those found by Baxter et al. [30] for bootstrap percolation (there, activation of a node requires *k* active neighbors).

Watts [5], proposed the first analytic solution for the TM, using percolation theory and generating functions to measure the size of the largest cluster of nodes requiring only one active neighbor to turn active (largest vulnerable cluster). The model applies to unweighted, undirected graphs with small clustering coefficient. In the infinite system size, when the vulnerable cluster percolates, there is a non-zero probability that a cascade will take over a large portion of the network (global cascade). A randomly selected initiator will activate the largest vulnerable cluster, if it is a part of the cluster or is one of its neighbors. Using this analytic method, Watts studied the regime for which global cascades are possible for one initiator, for different values of identical thresholds *ϕ*_0_ and average degree *z* of synthetic graphs. He found that, for ER graphs with *O*(1) initiator the criterion for global cascades is *z* < 1/*ϕ*_0_.

Gleeson and Cahalane [14] formulated an analytic approach for the TM with varying initiator sizes. Their work was inspired by the zero-temperature Random-Field Ising Model (RFIM) [31, 32], where the cascade size, the initiator size and the threshold distribution correspond to the magnetization, the external uniform field and the local quenched random fields of the RFIM. The main difference between the two models is that in the TM the activated nodes remain activated, while in the RFIM the spins may flip back to an inactive state. The analytic approach to the TM model is applicable to locally tree-like structures [14], such as ER graphs. The graph is considered an infinite-level tree with a level-by-level updating of the spread size, starting from the bottom of the tree.

In most of the past research, the cascade size has been thoroughly investigated for a identical threshold in the network [5, 10–13], or for a random threshold for each node [24, 25]. However, a model with identical thresholds does not capture the complex nature of social influencing when multiple initiators are present. The small scale experiment conducted by Latane [4] and more recently an online experiment by Centola [33] and a large online study on Facebook data [34] suggest that individuals have diverse thresholds for adopting a newly introduced opinion. Here, to capture the diversity of opinion adoption thresholds in a social influence context, we study the effect of heterogeneous thresholds on the cascade size under the TM for empirical and synthetic unweighted and undirected networks for randomly selected initiators.

## Materials and Methods

### Simulations of the Threshold Model

We assume that the thresholds are drawn from a truncated normal distribution with mean *ϕ*_0_ and standard deviation *σ*. The threshold *ϕ* of each node is limited to interval [0, 1], thus the mean threshold *ϕ*_0_ is also within this interval, and *σ* is in the range of [0, 0.288], boundaries of which correspond to the identical threshold and to the random threshold, respectively. Unlike, in the formulation of the threshold model in [14, 15], where thresholds drawn can be negative, allowing nodes to get spontaneously activated as innovators, and as a result randomizing the set of initiators, we are interested in the case where spread is initiated only with the insertion of randomly selected initiators in the network.

Once a threshold for each node is set, for the simulations, we randomly assign initiators one by one and measure the cascade size. We repeat this process by drawing thresholds from the same distribution. The final cascade size for each threshold distribution is obtained by averaging one thousand times on different threshold distribution draws and, for the synthetic graphs, different network realizations.

### Network Structures

The networks we use are undirected and unweighted. The synthetic networks used are Erdős-Rényi (ER) graphs and scale-free (SF) networks. For the generation of ER graphs [29] we used the *G*(*N*, *p*_ER_) model with *N* being the system size and *p*_*ER*_ the probability that a random node will be connected to any node in the graph. The probability *p*_*ER*_ is given by *p*_*ER*_ = *z*/(*N* − 1), where *z* is the nominal average degree in the network. We keep the average degree *z* = 10. For the generation of uncorrelated SF networks [35, 36] (*N* = 10^4^, *z* = 10, with power law constant *γ* = 3) we employ the configuration model [36, 37] with a structural cut-off, and a maximum possible node degree set to $\sqrt{N}$, using a high accuracy look-up table from [38].

The empirical networks used are a connected ego-network from a Facebook (FB) dataset, available from the Stanford Network Analysis Project (SNAP) [39] (system size *N* = 4048, average degree *z* = 43), and a high-school (HS) friendship network [40]. For the HS network, we only used the giant connected component of that network, with *N* = 921 and *z* = 5.96. The network contains two communities which are roughly equal in size (for more information on the two empirical networks see table in S1 Text). Although SF, FB, and HS networks are connected networks, the generated ER graphs may have a disconnected component with probably *e* ^−*z*^, which for *z* = 10 is approximately 0.000045.

### Tree-like approximation for the Threshold Model

For analytic methods, we apply Gleeson’s and Cahalane’s tree-like approximation for synthetic networks [14, 15]. The approximation is given by the following set of equations

Seq=p+1-p∑k=1∞Pk∑m=1kkmq∞m1-q∞k-mFmk (1)

qn+1=p+1-p∑k=1∞kzPk∑m=1k-1k-1mqnm1-qnk-m-1Fmk. (2)

In this approximation the graph is considered an infinite level tree. The spread diffuses level-by-level starting from the bottom of the tree. *q*_*n*_ is defined “as the conditional probability that a node on level *n* is active, conditioned on its parent on level *n* + 1 being inactive” and it is given by Eq (2). The final spread *S*_*eq*_ is given by Eq (1), and is measured at the top of the tree. The fraction of initially active nodes is given by *p*. In the bottom of the tree at level *n* = 0, the fraction of active nodes is only based on the initiators, thus *q*_0_ = *p*. The graph degree distribution is given by *P*_*k*_, which for an infinite size ER graph is given by *P*_*k*_ = (*z* ^*k*^ *e* ^−*z*^)/*k*!, where *z* is the average degree, while for SF networks it’s given by *P*_*k*_ ∼ *k* ^−*γ*^. $F\;(\frac{m}{k})$ is the cumulative probability that a node requires m or less active neighbors to get active, which depends on the assigned threshold distribution.

## Results

**Fig 1:**
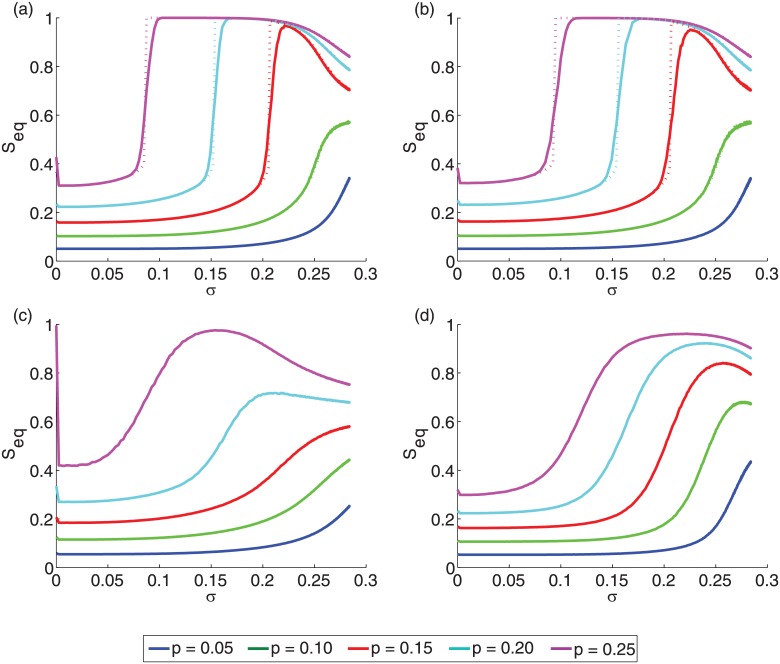
Behavior of the cascade size *S_eq_* at equilibrium for varying standard deviation *σ*. (a) ER graphs with *z* = 10 and *N* = 10^4^; (b) SF networks with *z* = 10, *γ* = 3, and *N* = 10^4^; (c) high-school network with *z* = 5.96 and *N* = 921; (d) facebook network with *z* = 43 and *N* = 4039. The mean threshold is *ϕ*_0_ = 0.50. The simulations are averaged over one thousand repetitions. (a) and (b) also show the analytic estimates (dotted lines) based on the tree-like approximation (see Materials and Methods) [14].

**Fig 2:**
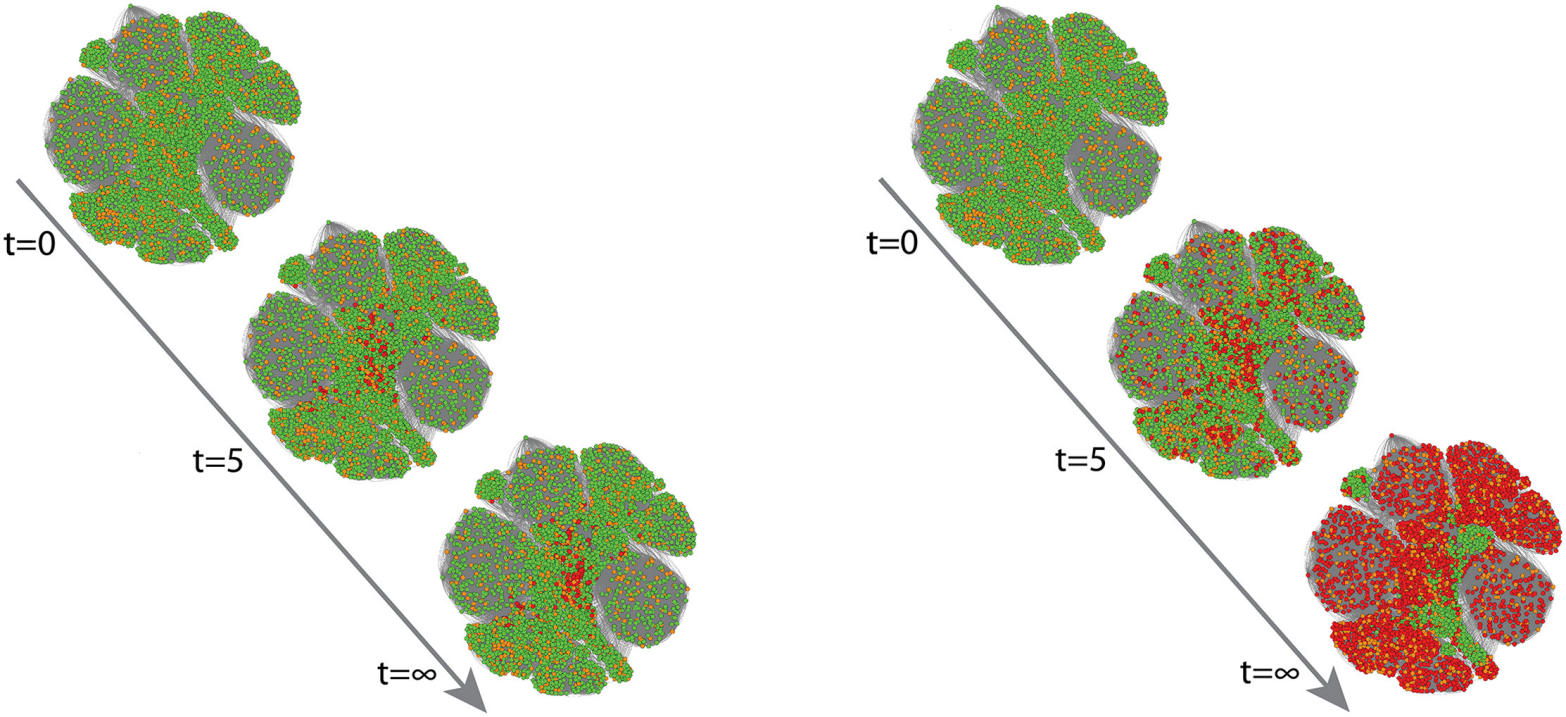
Visualization of the spread of opinion in the TM model on a facebook network with *z* = 43 and *N* = 4039. The fraction of the randomly selected initiators is *p* = 0.20. The mean threshold is *ϕ*_0_ = 0.50 while the standard deviation of the threshold is (a) *σ* = 0, (b) *σ* = 0.20. Inactive nodes, initiators, and active nodes (through spreading) are marked with green, orange, and red, respectively.

First, we examine the effect of the standard deviation *σ* on the cascade size *S*_*eq*_ (averaged) for a constant initiator fraction and constant mean threshold *ϕ*_0_ (Fig 1). As *σ* increases so does a fraction of nodes whose threshold is far from the average causing a twofold effect. Of nodes far from average, the ones with thresholds below average are easily activated while those with thresholds above average are increasingly difficult to activate. Thus, when the initiator fraction is small, the cascade size *S*_*eq*_ is monotonically increasing since the presence of larger fraction of low threshold nodes facilitate the spread. However, when the initiator fraction are large, the increase in low threshold nodes helps a little since they are likely to be already activated without the increase in *σ*, but presence of additional high threshold nodes arrest the spread. This trade-off gives rise to the non-monotonic behavior seen in Fig 1, which is apparent for different types of networks. Depending on the network structure and size of the initiators, the standard deviation *σ* for which the spread is optimal varies. A visualization (Fig 2) shows time steps of the spread on a random selection of initiators with *p* = 0.20 in the FB network. For the same set of initiators, the spread for large sigma (*σ* = 0.20) is much higher than for identical thresholds (*σ* = 0.00). Interestingly, in the vicinity of *σ* ∼ 0 the sharp decrease in the cascade size *S*_*eq*_ occurs because with non-zero *σ*, approximately half of the nodes acquire a threshold higher than *ϕ*_0_ = 0.50. For all the nodes with threshold *ϕ* > *ϕ*_0_ with even degree, even the slightest non-zero *σ* value will increase the number of active neighbors by one, thus making cascades less likely to occur. Finally, for ER graphs [Fig 1(a)] and SF networks [Fig 1(b)] the analytic estimates are in good agreement with the simulations.

**Fig 3:**
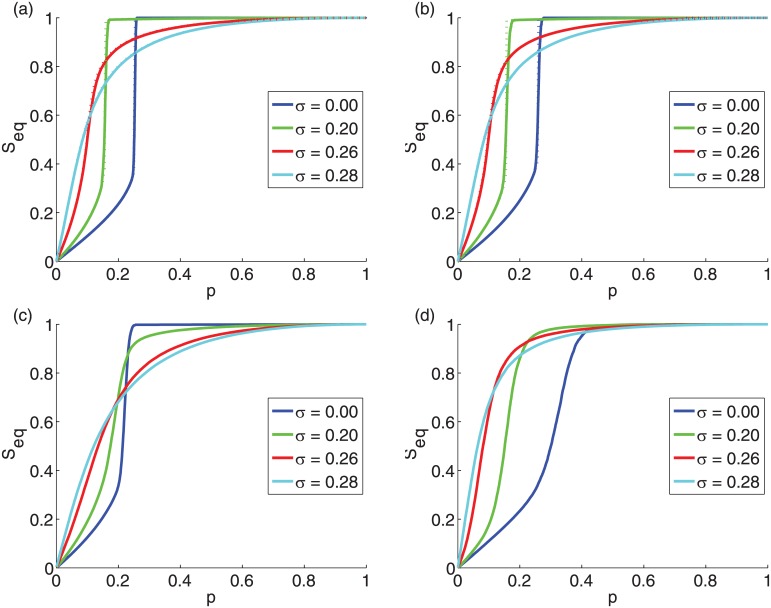
Behavior of the cascade size *S_eq_* at equilibrium vs. the initiator fraction *p*. The networks are the same as in Fig 1: (a) ER graphs with *z* = 10 and *N* = 10^4^; (b) SF networks with *z* = 10, *γ* = 3, and *N* = 10^4^; (c) high-school network with *z* = 5.96 and *N* = 921; (d) facebook network with *z* = 43 and *N* = 4039. The mean threshold is *ϕ*_0_ = 0.50. (a) and (b) also shows the analytic estimates (dotted lines) based on the tree-like approximation (see Materials and Methods) [14].

In Fig 3, the cascade size *S*_*eq*_ is plotted for varying initiator sizes *p* for the same networks as in Fig 1. As the initiator fraction increases, for small enough *σ* there is a transition from small local cascades to large global cascades, which, for synthetic graphs is a discontinuous phase transition [Fig 3(a) and 3(b)]. However, the line of the average cascade size *S*_*eq*_ appears smooth even in the presence of a discontinuous phase transition, because for each repetition the point of the discontinuous phase transition varies slightly. With increasing *σ* the initiator fraction for which the transition occurs is reduced, while for the synthetic graphs the spread size still exhibits a discontinuous phase transition. With largely diverse thresholds we find that a critical initiator size beyond which cascades become global ceases to exist and the tipping-point behavior of the social influencing process disappears and is replaced by a smooth crossover governed by the size of initiator set. This property can be important, for example, for a company’s marketing strategy of a new product. If the threshold distribution is narrow enough, unless a critical initiator fraction is reached, there is a marginal local spread on a few of the first or second neighbor friends of the initiators. On the other hand, if the threshold distribution is wide, there is a significant spread. For the uniform random threshold distribution each addition of initiators has a reduced contribution to the cascade size as predicted by the submodularity property of the TM [24].

**Fig 4:**
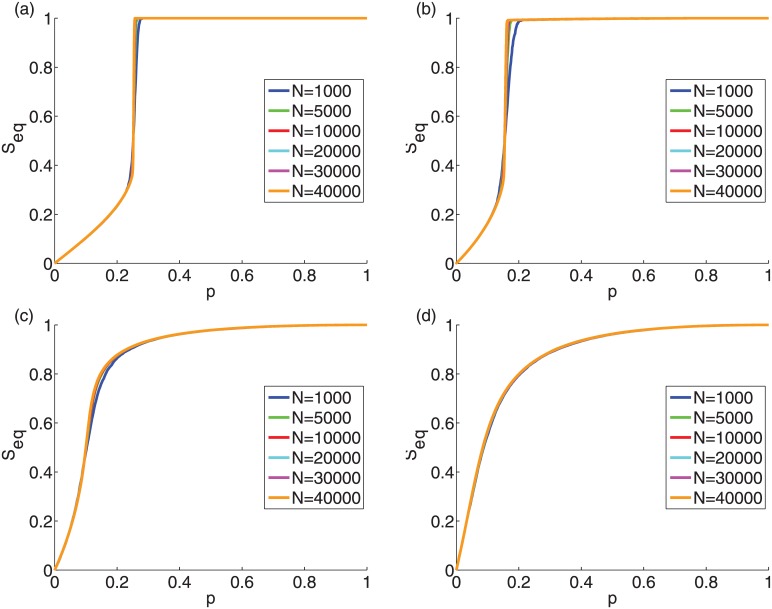
Finite-size behavior of the final cascade size *S_eq_* vs. the initiator fraction *p* for ER graphs with average degree *z* = 10. The mean threshold is *ϕ*_0_ = 0.50 while the standard deviation of the threshold is (a) *σ* = 0.00, (b) *σ* = 0.20, (c) *σ* = 0.26 and (d) *σ* = 0.28.

**Fig 5:**
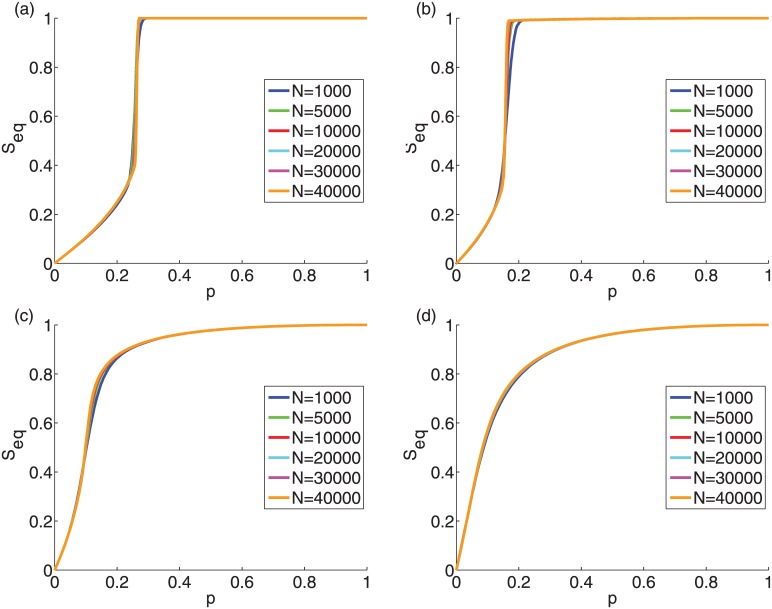
Finite-size behavior of the final cascade size *S_eq_* at vs. the initiator fraction *p* for SF networks with *z* = 10 and *γ* = 3. The mean threshold is *ϕ*_0_ = 0.50 while the standard deviation of the threshold is (a) *σ* = 0.00, (b) *σ* = 0.20, (c) *σ* = 0.26, (d) *σ* = 0.28.

In Figs 4 and 5 we show that the behavior of the cascade size is largely independent of the system size *N* for any threshold distribution with the same degree distribution, for ER graphs and SF networks, respectively. We observe that with increasing system size *N* the cascade size *S*_*eq*_ is asymptotically converging.

**Fig 6:**
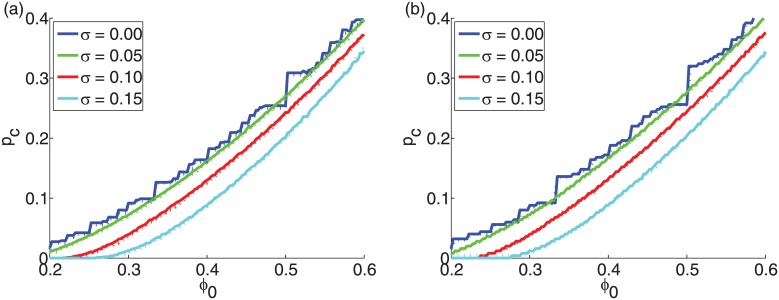
Critical initiator fraction *p_c_* vs. mean threshold *ϕ*_0_. (a) ER graphs and (b) SF networks with *γ* = 3 with average degree *z* = 10 and system size *N* = 10^4^. An initiator size above the *p*_*c*_ line leads to global cascades. The analytic estimates (dotted lines) are based on the tree-like approximation [14] (see Materials and Methods).

We record the critical initiator fraction *p*_*c*_ for which a discontinuous phase transition occurs for varying mean threshold *ϕ*_0_ (Fig 6). For the measurement of *p*_*c*_, first we calculated the derivative of the *S*_*eq*_ from Fig 3 with respect to the initiator fraction *p*. The position of maximum of the derivative yields the *p*_*c*_, in other words, *p*_*c*_ = arg max_*p*_(*dS*_*eq*_(*p*)/*dp*). We used the same method for the calculation of the respective analytic estimates. We confine the threshold distribution for up to *σ* = 0.15 to assess if there is a discontinuous phase transition with increasing initiators. Above each *p*_*c*_ line global cascades occur. The value of *p*_*c*_ decreases with increasing *σ*. For identical thresholds *ϕ*_0_ (in blue), the *p*_*c*_ line has some sharp jumps, for example at *ϕ*_0_ equal to 0.50, 0.33, and 0.25 (Fig 6). These jumps are artifacts of the discrete steps of the degree distribution in the presence of a unique threshold for all the nodes. In particular, microscopically, the number of active neighbors required for a node to turn active increases by integer values. For example, for a node with degree 10 and 0.40 < *ϕ* ≤ 0.50, that number is 5. For identical thresholds in the network, the cumulative effect of these integer steps gives rise to the jumps exhibited by the *p*_*c*_(*ϕ*_0_) curves (Fig 6). Interestingly, this effect also shows in Fig 1, where for large enough initiator fractions (i.e., *p* = 0.25 or higher) the cascade size drops abruptly as *σ* is increased from zero to small values. For nodes with mean threshold *ϕ*_0_ = 0.50, even the smallest non zero increase on the standard deviation *σ* results in approximately half of the nodes having threshold larger than *ϕ*_0_ = 0.50. The *p*_*c*_ lines are lower for the ER graph compared to the SF networks because of the importance of a randomly selected very high degree node in SF networks can have on the spread. Our results obtained from simulations are in agreement with the analytic estimates.

**Fig 7:**
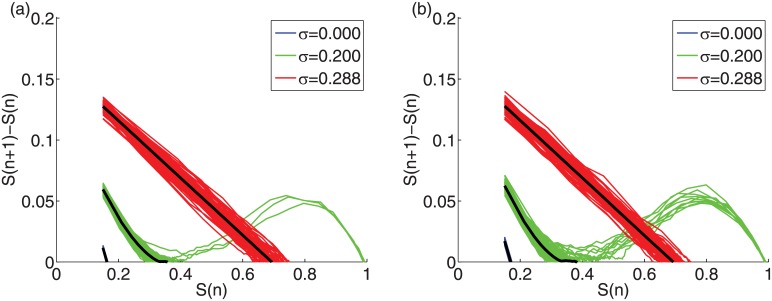
Phase-space diagrams for a constant initiator fraction *p* = 0.15. and various standard deviations *σ* = 0 (blue), *σ* = 0.2 (green), *σ* = 0.288 (red) for (a) ER graphs and (b) SF networks with *γ* = 3, with *z* = 10 and *N* = 10^4^. The colored lines refer to a hundred independent repetitions, while the black lines are their averages.

To further understand the effect of the standard deviation *σ*, we study the dynamics of the spread for synchronous updating of the nodes. In phase-space, as shown in Fig 7, the difference *ΔS*(*n* + 1) − *ΔS*(*n*) defines the number of nodes activated from time step *n* to *n* + 1. The dynamic spread in the TM is deterministic and evolves in one direction, hence, the spread stops when the change on the cascade size (*Y*-axis) reaches zero. Accordingly, the value of the cascade size in the steady state is indicated on the *X*-axis. When cascades are not possible, the spread rate decreases monotonically. However, when cascades are possible then for up to some *σ* the change is non-monotonic and the fractions of nodes in cascades reach almost one. But as *σ*’s grow larger and larger, these fractions stop growing farther and stay farther from one. When *σ* approaches the standard deviation of uniform distribution the shape of the lines decreases linearly. Interestingly, similar behavior is observed for the FB and HS networks as well.

### Closed-form analytic estimate for the uniform threshold distribution

**Fig 8:**
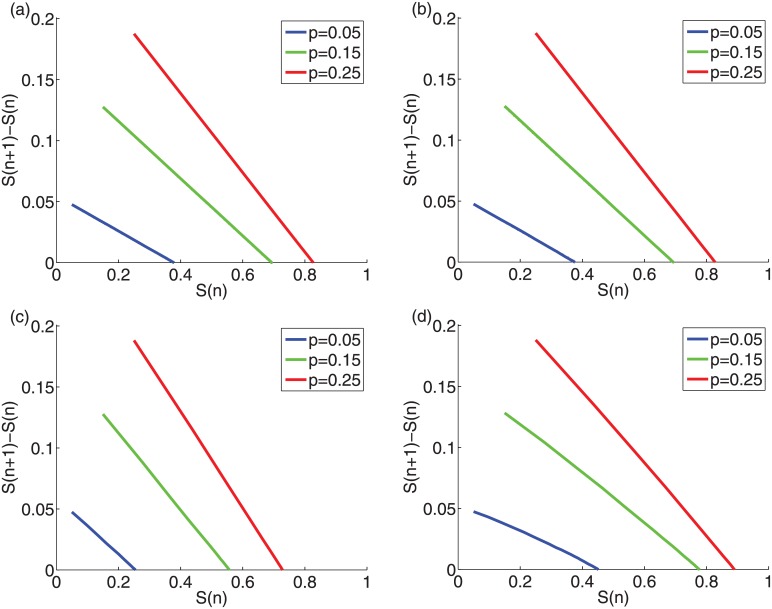
Phase-space diagrams for the uniform random threshold distribution (*σ* = 0.288). for various initiator fractions *p* = 0.05 (blue), *p* = 0.15 (red) and *p* = 0.25 (green) for (a) ER graphs, (b) SF networks, (c) high-school network, and (d) facebook network as in Fig 1. The solid lines and dotted lines (complete overlap) correspond to the simulations and to the closed-form analytic estimates [Eq (2)], respectively.

For a uniform threshold distribution the phase-space line decreases linearly for any initiator fraction for synthetic graphs and almost linearly for the empirical networks (Fig 8). In addition, we show for this threshold distribution, using Gleeson’s and Cahalane’s analytical methods, that the phase-space line has a closed form and is linearly decreasing. The extended proof of this is shown in S1 Text. For a uniform threshold distribution the iterative formula in Eq (2) of the analytic approximation yields the following closed-form solution

$$q_{n+1}=p+bq_{n},$$ (3)

with $b=(1-p)\;\frac{1}{z}\;(z-1+P_{0})$. The solution of the above iterative equation with the initial condition *q*_0_ = *p*, is

$$q_{n}=p\frac{1-b^{n+1}}{1-b}.$$ (4)

According to [16], the spread at level *n*+1 is given by

Sn+1=h(qn)=p+1-p∑k=1∞Pk∑m=1kkmqnm1-qnk-mFmk, (5)

which, in the case of a uniform distribution of thresholds (S1 Text) simplifies to

$$S_{n+1}=p+cq_{n},$$ (6)

with *c* = (1 − *p*)(1 − *P*_0_), where the initial spread is *S*_0_ = *p*. Using the above Eq and Eq (3) we can calculate (S1 Text) the formula for the phase-space diagram

$$S_{n+1}-S_{n}=cp-\left(1-b\right)p-\left(1-b\right)S_{n}$$ (7)

The above Eq is the closed form phase-space line of Fig 8. On the other hand, at the equilibrium (as *n* → ∞) the spread size in Eq 6 becomes

$$S_{eq}=p+cq_{\infty },$$ (8)

with $q_{\infty }=p\frac{1}{1-b}$ (S1 Text). Note that in this approximation for uniform threshold distribution, the size of the final cascade for uncorrelated networks does not dependent on the details of the degree distribution, it only depends on the average degree *z*. In addition, it is easy to show that the derivative of the final cascade size [Eq (8)] with respect to the initiator size *p* is monotonically decreasing, in agreement with the submodularity property of the TM for the uniform threshold distribution [24].

### Discontinuous phase transitions in the threshold model

To further understand the final cascade size behavior at the critical point for synthetic graphs, we are examining the system size dependence. The spread size at the equilibrium is independent of the method of the insertion of initiators, e.g., it does not matter whether the addition occurs in fractions or by individual addition of initiators. Using Monte-Carlo simulations, Singh [10] showed that the average cascade size is largely independent of the system size for the same initiator fraction for an identical threshold for ER graphs with unique degree distribution. We use the same approach to show that this is true for other threshold distributions for ER graphs (Fig 4) and SF networks (Fig 5). These results indicate that given an initiator fraction *p*_0_ and an average cascade size *S*_*eq*_(*p*_0_), the addition of another initiator fraction *p*_1_ will cause the same change *ΔS* = *S*_*eq*_(*p*_0_+*p*_1_) − *S*_*eq*_(*p*_0_) in the average cascade size *S*_*eq*_, largely independently of the system size, for large system sizes, for the same input degree and threshold distributions.

**Fig 9:**
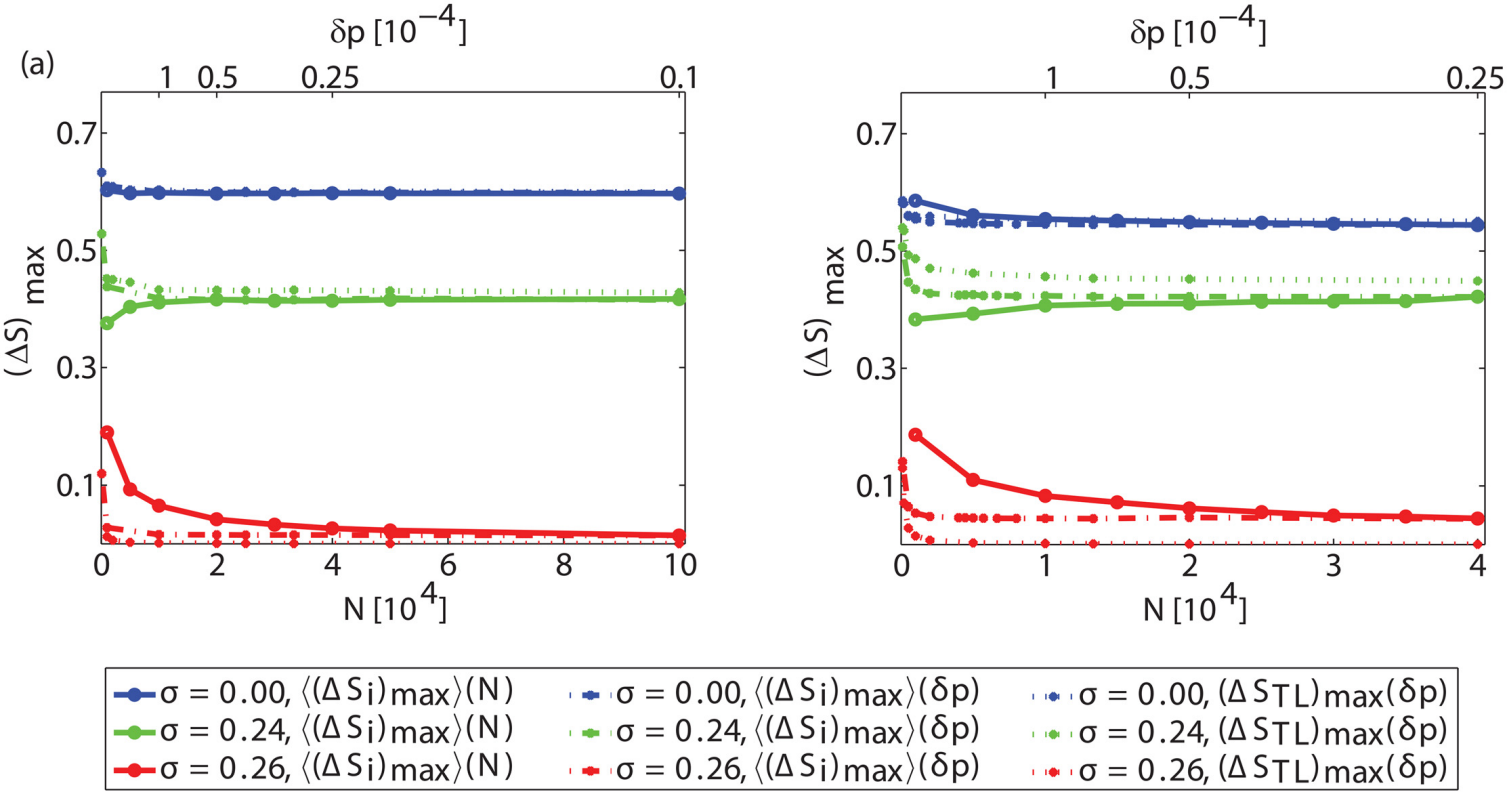
Maximum contribution of initiators to the cascade size for various *σ* values. (a) for ER graphs and (b) for SF networks with *γ* = 3, for *z* = 10. Solid lines: 〈(*ΔS*_*i*_)_max_〉(*N*) of O(1) initiator with one-by-one addition of initiators for varying system sizes (bottom x-axis). Dashed lines: 〈(*ΔS*_*i*_)_max_〉(*δp*) for various initiator fractions (top x-axis) for a constant system size *N* = 10^5^. Dotted lines: (*ΔS*_*TL*_)_max_(*δp*) for various initiator fractions (top x-axis) for the TL approximation. The mean threshold is kept at *ϕ*_0_ = 0.50 in all cases.

Our analysis so far focused on the cascade size at the steady state *S*_*eq*_ averaged over many realizations of networks, threshold values and assignment of initiators (Figs 4 and 5). To verify the presence and nature of phase transitions, we follow the approach presented in [30]. We start by measuring the increase of the cascade size of each sample in response to the one-by-one addition of initiators. If a discontinuous phase transition arises, at the critical point, the increase of the cascade size should remain constant and independent of the system size. To investigate this, let *v* be the current size of initiator set. For a given sample *i*, let $\Delta S_{i}=S_{i}\left(\frac{v+1}{N}\right)-S_{i}\left(\frac{v}{N}\right)$ denote the increase in the cascade size caused by the addition of a single randomly selected initiator to the current initiator set. Let (*ΔS*_*i*_)_max_(*N*) be the maximum value of *ΔS*_*i*_(*N*) for all initiator sets of size $\frac{v}{N}$. Then, varying *σ*, we study how (*ΔS*_*i*_)_max_(*N*) averaged over one thousand repetitions depends on the system size *N* (Fig 9) (solid lines). We observe that for the plotted cases with *σ* = 0.00 and *σ* = 0.24, 〈(*ΔS*_*i*_)_max_〉(*N*) is independent of the system size. Moreover, the contribution of the rest of the initiators to the cascade tends to zero in the limit of infinite system size. However, for *σ* = 0.26, 〈(*ΔS*_*i*_)_max_〉(*N*) decreases with the system size, indicating the absence of a discontinuous phase transition in the infinite system-size limit. Thus, there appears a qualitative change somewhere between *σ* = 0.24–0.26.

A similar analysis can be applied to the analytical estimation, with the tree-like approximation, of the increase in the cascade size (*ΔS*_*TL*_)_*max*_(*δp*) with a marginal addition of initiators. However, since the analytical estimation is set for an infinite system size, the one-by-one addition of initiators on larger and larger system sizes is not possible. Hence, we insert smaller and smaller fractions of initiators *δp*. In Fig 9 the top X axis is the fractional step increase of the number of initiators. For consistency, we include the corresponding increase in the cascade size 〈(*ΔS*_*i*_)_max_〉(*δp*) that *δp*, a fractional step increase of the number of initiators, measured through simulations. In this case, the minimum possible fraction of initiators is *δp* = 1/*N*. We observe, that the results for the one-by-one addition of initiators with varying systems through simulations, agree with those for the fractional increase of an infinite system size with varying *δp*. We conclude that it is between *σ* = 0.24–0.26 (for *ϕ*_0_ = 0.50) where the discontinuous phase transitions cease to emerge in the thermodynamic limit.

## Discussion

Past experimental online studies [4, 33, 34] indicate the existence of diverse adoption thresholds of individuals in social networks. Prompted by this observation, we studied the impact of diversity of thresholds in spreading a new opinion, by intuitively assuming that the adoption thresholds are drawn from a truncated normal distribution. We explored this impact by using the threshold model, a reinforcement model which has lately drawn significant attention in the scientific community. We showed that in the presence of a small spread (standard deviation) of the threshold distribution in a network, unless a critical initiator fraction is reached, the impact of the randomly selected initiators is small. Furthermore, we showed that, when discontinuous transitions in cascade size are possible for synthetic graphs, the addition of a single randomly-selected initiator can have a significant (global) impact on the final cascade size, i.e., the manifestation of the tipping point. However, with a sufficiently large spread in the individual thresholds (with the same mean), the cascade size exhibits a smooth transition, where the impact of each added initiator is reduced by the current size of the initiator set. Finally, we showed that in the case of a uniform threshold distribution, the spreading rate is linearly decreasing with the spread size for synthetic graphs and close to linearly decreasing for empirical graphs. In summary, our results indicate that information on the diversity of the thresholds is critically important for the understanding of the behavior of cascades in threshold-limited social contagion with multiple initiators. Most importantly, sufficiently large spread in the individual thresholds can change not only the quantitative aspects of triggering global cascades, but also the qualitative behavior of the system: the cascade size exhibits a smooth change (as opposed to a discontinuous jump) as a function of the fraction of initiators.

## Supporting Information

S1 Text Includes basic statistics of the two empirical networks used and the closed-form analytical estimate for the case of the uniform distribution of thresholds. (PDF)
