# Correction: The Impact of Heterogeneous Thresholds on Social Contagion with Multiple Initiators

**DOI:** 10.1371/journal.pone.0154980

**Published:** 2016-04-28

**Authors:** Panagiotis D. Karampourniotis, Sameet Sreenivasan, Boleslaw K. Szymanski, Gyorgy Korniss

The following information is missing from the Funding section: Funding was also provided by the National Science Centre, decision no. DEC-2013/09/B/ST6/02317.

Additionally, one of the affiliations for the third author is not indicated. Boleslaw K. Szymanski is also affiliated with the Wroclaw University of Technology, Poland.

## References

[1] Karampourniotis PD, Sreenivasan S, Szymanski BK, Korniss G (2015) The Impact of Heterogeneous Thresholds on Social Contagion with Multiple Initiators. PLoS ONE 10(11): e0143020 doi: 10.1371/journal.pone.0143020 26571486 10.1371/journal.pone.0143020 PMC4646465

