# Supplementary material for: The Impact of Heterogeneous Thresholds on Social Contagion with Multiple Initiators
Source: PLoS One. 2015 Nov 16;10(11):e0143020. doi: 10.1371/journal.pone.0143020 (PMC4646465; doi:10.1371/journal.pone.0143020)
Supplement: S1 Text — (PDF) [file pone.0143020.s001.pdf]

The Impact of Heterogeneous Thresholds on Social Contagion with Multiple Initiators

Supporting Information: S1 text

PD Karampourniotis[1,2]*, S Sreenivasan[1,2], BK Szymanski[2,3], G Korniss[1,2]

October 5, 2015

1 Department of Physics, Applied Physics, and Astronomy, Rensselaer Polytechnic Institute, 110 8th Street, Troy, NY, 12180-3590 USA
2 Social Cognitive Networks Academic Research Center, Rensselaer Polytechnic Institute, 110 8th Street, Troy, NY, 12180-3590 USA
3 Department of Computer Science, Rensselaer Polytechnic Institute, 110 8th Street, Troy, NY, 12180-3590 USA

Table 1: Basic statistics of the two empirical networks used

Network	Type	N	m	z	α	d	C_1	C_2	ρ
Facebok	Undirected	4039	88234	43.691	1.72	8	0.2647	0.6055	0.5432
High School	Undirected	921	2745	5.9674	3.30	12	0.0521	0.1254	0.2817

Table 1: Basic statistics of the two empirical networks used. The properties measured are: the type of network (directed or undirected), total number of nodes N, total number of edges m, average degree z, power law coefficient α, network diameter d, fraction of closed triangles C_1, average clustering coefficient C_2, Spearman's assortativity ρ.

Closed-form analytical estimate

Here, we show explicitly the derivation of the closed form equation of the treelike approximation [1–2] of the fraction S_n of active nodes at level n on Eq. (6) in the main text. According to [2] the level (or time) dependent evolution of the fraction q_{n+1} of nodes with inactive parents at level $n+1$ for synchronous updating of the nodes is given by

$$q_{n+1} = g(q_n) = p + (1-p)\sum_{k=1}^{\infty}\frac{k}{z}P_k\sum_{m=1}^{k-1}\binom{k-1}{m}q_n^m\,(1-q_n)^{k-m-1}\,F\left(\frac{m}{k}\right), \tag{S1}$$

*E-mail: karamp@rpi.edu

and the fraction of active nodes at level $n+1$ is given by

$$S_{n+1} = h(q_n) = p + (1-p) \sum_{k=1}^{\infty} P_k \sum_{m=1}^{k} \binom{k}{m} q_n^m (1-q_n)^{k-m} F\left(\frac{m}{k}\right). \tag{S2}$$

The replacement of the cumulative probability function $F\left(\frac{m}{k}\right)$ in the particular case of a uniform distribution of thresholds in the above two equations yields the closed form solution. Let a node i have degree k and an assigned threshold ϕ. Vulnerability l is the absolute number of active neigbhors required for node i to get activated, and it is given by $l = \text{ceil}(\phi \times k)$. The cumulative probability distribution $F\left(\frac{m}{k}\right)$ of nodes with degree k, having vulnerability less or equal to m, is given by $F\left(\frac{m}{k}\right) = \sum_{k=1}^{m} r_{l,k}$, where $r_{l,k}$ is the probability that a node has vulnerability l, conditioned that it has degree k. For a uniform threshold distribution the probability that a node has vulnerability l, conditioned that it has degree k, is $r_{(l,k)} = 1/k$. For example, a node with degree $k = 2$ will have vulnerability $l = 1$, with probability $r_{(1,2)} = 1/2$ and vulnerability $l = 2$ with probability $r_{(2,2)} = 1/2$. Thus, the fraction $F\left(\frac{m}{k}\right)$ of nodes that have vulnerability m or less conditioned that they have degree k for the uniform random threshold distribution is given by

$$F\left(\frac{m}{k}\right) = \sum_{k=1}^{m} r_{l,k} = \sum_{k=1}^{m} \frac{1}{k} = \frac{m}{k}. \tag{S3}$$

Now, replacing Eq. S3 in Eq. S1 we show the linear relationship between the fraction q_{n+1} of nodes with inactive parents at level $n+1$ with the fraction q_n at the previous level n of the approximated tree for networks with uniform distribution of thresholds (see Eq (3) in the main text). So,

$$q_{n+1} = p + (1-p) \sum_{k=1}^{\infty} \frac{k}{z} P_k \sum_{m=1}^{k-1} \binom{k-1}{m} q_n^m (1-q_n)^{k-1-m} \frac{m}{k}, \tag{S4}$$

which simplifies to

$$q_{n+1} = p + (1-p) \frac{1}{z} \sum_{k=1}^{\infty} P_k \sum_{m=1}^{k-1} \binom{k-1}{m} q_n^m (1-q_n)^{k-1-m} m. \tag{S5}$$

However,

$$\sum_{m=1}^{k} \binom{k}{m} q_n^m (1-q_n)^{k-m} m = \sum_{m=0}^{k} \binom{k}{m} q_n^m (1-q_n)^{k-m} m, \tag{S6}$$

where the right hand of the equation is the mean of the binomial distribution, and it is given by kq_n [3], thus

$$\sum_{m=1}^{k-1} \binom{k-1}{m} q_n^m (1-q_n)^{k-1-m} m = (k-1)\, q_n \tag{S7}$$

Using the above equation in Eq. (S5) yields

$$q_{n+1} = p + (1-p) \frac{1}{z} \sum_{k=1}^{\infty} P_k (k-1)\, q_n, \tag{S8}$$

which can be rewritten as

$$q_{n+1} = p + (1-p) \frac{1}{z} \left(\sum_{k=0}^{\infty} P_k (k-1) + P_0 \right) q_n. \tag{S9}$$

Since the average degree is given by $z = \sum_{k=0}^{\infty} kP_k$, the above equation becomes

$$q_{n+1} = p + (1-p)\frac{1}{z}(z - 1 + P_0)\, q_n. \tag{S10}$$

which can be rewritten as

$$q_{n+1} = p + bq_n, \tag{S11}$$

with $b = (1-p)\frac{1}{z}(z - 1 + P_0)$. The solution of the above equation with initial condition $q_0 = p$ is

$$q_n = p\frac{1 - b^{n+1}}{1 - b} \tag{S12}$$

Similarly, replacing $F\left(\frac{m}{k}\right)$ in S2 by the right hand side of Eq. (S3), the analytic approximation yields

$$S_{n+1} = p + (1-p)\sum_{k=1}^{\infty} P_k \sum_{m=1}^{k} \binom{k}{m} q_n^m (1 - q_n)^{k-m}\frac{m}{k}. \tag{S13}$$

Using again the property of the mean of the binomial distribution the above equation reduces to

$$S_{n+1} = p + (1-p)\sum_{k=1}^{\infty} P_k \frac{1}{k}(kq_n), \tag{S14}$$

which yields

$$S_{n+1} = p + (1-p)\, q_n \sum_{k=1}^{\infty} P_k. \tag{S15}$$

Thus, the closed form solution of cascade size at level $n+1$ is given by

$$S_{n+1} = p + cq_n, \tag{S16}$$

with $c = (1-p)(1-P_0)$. Subtracting S_n from both parts of the above equation and combining it with Eq. S11 we get

$$S_{n+1} - S_n = c\,(q_n - q_{n-1}). \tag{S17}$$

Substituting $q_n = p + bq_{n-1}$ from Eq. S11 into the above equation yields

$$S_{n+1} - S_n = c\,(p + (b-1)q_{n-1}). \tag{S18}$$

Solving Eq. S16 for q_{n-1} at level $n-1$ and substituting to the above equation yields

$$S_{n+1} - S_n = c\left(p + (b-1)\left(\frac{S_n - p}{c}\right)\right). \tag{S19}$$

Expansion of the above equation yields to the closed form phase-space equation at Eq. (6) in the main text

$$S_{n+1} - S_n = cp - (1-b)p - (1-b)S_n. \tag{S20}$$

Now, going back to the calculation of S_{n+1} at Eq. S16, substituting q_n with the right part of Eq. S12 yields

$$S_{n+1} = p + cp\frac{b^{n+1} - 1}{b - 1}, \tag{S21}$$

where the cascade size S_0 at level $n = 0$ is just the fraction of the initiators, $S_0 = p$. On the other hand, in the equilibrium state (as $n \to \infty$) the cascade size S_{eq} is given by

$$S_{eq} = p + cp\frac{1}{1 - b}, \tag{S22}$$

since $0 \leq b < 1$. Interestingly, the final cascade size doesn't depend for uncorrelated networks on the degree distribution, but only on the average degree z.

References

[1] Gleeson JP, Cahalane DJ. Seed size strongly affects cascades on random networks. Phys. Rev. E. 2007; 75: 056103. doi: 10.1103/PhysRevE.75.056103

[2] Gleeson JP. Cascades on correlated and modular random networks. Phys Rev E. 2008; 77: 046117. doi: 10.1103/PhysRevE.77.046117

[3] Grimmett G, Welsh D. Probability: An Introduction. Oxford: Oxford University Press; 2014.
